# Astrocyte Networks as Therapeutic Targets in Glaucomatous Neurodegeneration

**DOI:** 10.3390/cells10061368

**Published:** 2021-06-02

**Authors:** Andrew M. Boal, Michael L. Risner, Melissa L. Cooper, Lauren K. Wareham, David J. Calkins

**Affiliations:** 1 Department of Ophthalmology and Visual Sciences, Vanderbilt Eye Institute, Vanderbilt University Medical Center, 1161 21st Ave S, AA7103D Medical Center North, Nashville, TN 37232-0654, USA; andrew.m.boal@vanderbilt.edu (A.M.B.); michael.l.risner@vumc.org (M.L.R.); lauren.wareham@vumc.org (L.K.W.); 2 Skirball Institute for Biomolecular Medicine, NYU Langone Medical Center, New York, NY 10016, USA; melissa.cooper@nyulangone.org; 3 Neuroscience Institute, NYU Langone Medical Center, New York, NY 10016, USA

**Keywords:** astrocyte, connexin-43, glaucoma, gap junction

## Abstract

Astrocytes are intimately involved in the response to neurodegenerative stress and have become an attractive target for the development of neuroprotective therapies. However, studies often focus on astrocytes as single-cell units. Astrocytes are densely interconnected by gap junctions that are composed primarily of the protein connexin-43 (Cx43) and can function as a broader network of cells. Such networks contribute to a number of important processes, including metabolite distribution and extracellular ionic buffering, and are likely to play an important role in the progression of neurodegenerative disease. This review will focus on the pro-degenerative and pro-survival influence of astrocyte Cx43 in disease progression, with a focus on the roles of gap junctions and hemichannels in the spread of degenerative stress. Finally, we will highlight the specific evidence for targeting these networks in the treatment of glaucomatous neurodegeneration and other optic neuropathies.

## 1. Introduction

Glaucoma is an age-related neurodegenerative disease that causes irreversible vision loss through the degeneration of retinal ganglion cells (RGCs) and their axons. By the year 2040 an estimated 100 million people world-wide will be affected by glaucoma [1]. Although aging is the primary risk factor for glaucoma, sensitivity to intraocular pressure (IOP) remains the only clinically modifiable risk factor. Stress caused by sensitivity to IOP is conveyed at the optic nerve head, which is composed of vulnerable unmyelinated RGC axons and a complex network of glial cells and extracellular matrix proteins. Reducing IOP even well below normal levels using hypotensive therapies often provides better prognosis by slowing progression of the disease [2], indicating optic nerve head tissue is more sensitive to the strain imposed by elevations in IOP. However, many patients continue to lose vision despite significant IOP control [3]. For these patients, nothing can be done to preserve vision because effective neuroprotective treatments do not exist. Furthermore, advances in the field are allowing for earlier diagnosis of glaucoma, at stages where neuroprotective intervention could have a greater impact on visual outcomes [4]. For these reasons, recent glaucoma research has focused on defining mechanisms of pathogenesis as well as developing and testing cell-based treatments that protect visual neurons.

IOP-related stress is conveyed by cellular mechanisms that pique RGC axons within the optic nerve head [5,6,7]. Thus, the first cellular responders to stress likely reside in the optic nerve head and represent primary targets for treatment. In humans, the optic nerve head consists of unmyelinated axons passing through a dense network of transversely oriented astrocytes, within a network of collagenous beams called the lamina cribrosa [8]. The rodent optic nerve head does not contain a true lamina cribrosa, though it does have a well-structured network of astrocytes that envelop RGC axons [9].

Astrocytes are promising targets for neuroprotective therapies. This premise is based on their location within the optic nerve head and their intrinsic neural support functions [10,11,12]. The potential neuroprotective influence of astrocytes on neurons is broad because astrocytes are densely interconnected via gap junctions. Connections between gap junctions allow astrocytes to function as a far-reaching syncytium, rather than being isolated as single cell units. Through these networks, astrocytes accomplish diverse tasks such as providing metabolic substrates in the form of lactate to neurons and regulating the extracellular ion gradients through spatial buffering.

Gap junctions directly connect neighboring cells, conducting inter-cell communication through electrical currents and the direct passage of small molecules. Gap junctions are formed from a large diversity of connexin (Cx) proteins. These proteins are assembled as complexes of six subunits, which are known as connexons. The connexons are inserted into the cell membrane and can then link adjacent cells via three main types of gap junction connections. First, gap junctions may be formed by two connexons both consisting of the same proteins (homotypic). Second, gap junctions may form between two distinct connexons, where each contains six of the same Cx proteins (heterotypic). Third, the composition of each individual connexon may be mixed (heteromeric) and form any variety of connection (for review see [13]).

Connexins are expressed by a variety of cells across different tissues. Within the central nervous system (CNS), connexins are expressed widely by glial cells and a subset of neurons [14]. Macroglia, predominantly astrocytes and oligodendrocytes, have strong connexin expression and form gap junction networks that allow them to function as a “panglial syncytium” (for a detailed review of glial connexins, see [15]). There is a diversity in connexin type and distribution across this glial network. Such diversity necessitates a detailed understanding of the connexin makeup for targeted therapeutic intervention.

**Figure 1:**
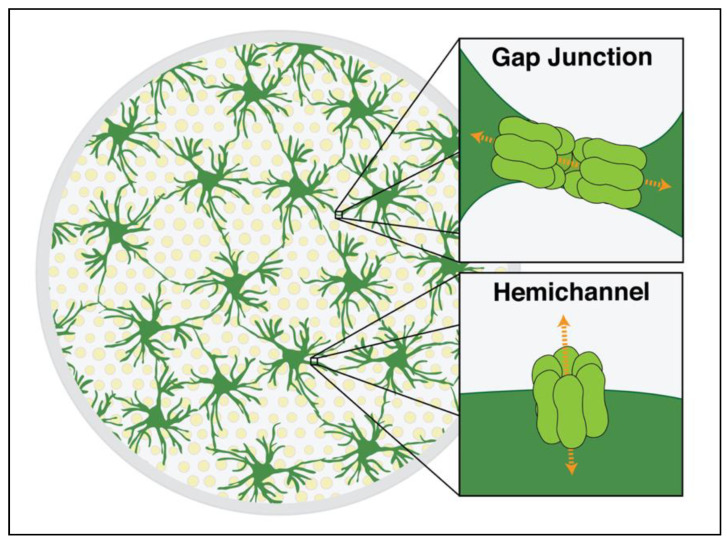
Schematic cross section of the rodent optic nerve head demonstrating Cx43 gap junctions and hemichannels. Optic nerve astrocytes (dark green) are arranged in a network that lies perpendicular to the direction of axons (yellow). These cells are interconnected via gap junctions composed of two adjoining hexamers of Cx43 (light green), as shown in the upper image inset. The junctions allow for electrical coupling and the nonselective passage of molecules up to ~1.2 kilodaltons in size from cell to cell. A single hexamer can also be unopposed on the astrocyte membrane, forming a hemichannel (lower inset). These can allow direct communication between the cytoplasmic and extracellular spaces.

Astrocytes in both the human and rodent CNS predominately express Cx30 and Cx43, which are heterogeneously distributed in different brain regions [16]. For example, the ratio of Cx30 to Cx43 is higher in the thalamus than other regions, such as the cortex [17]. Conversely, Cx43 dominates white matter tracts, such as in the optic nerve [18]. The dense network of astrocytes in the optic nerve head is highly linked by Cx43 gap junctions (Figure 1). Connections between two astrocytes are mediated by homotypic gap junctions (either Cx30/Cx30 or Cx43/Cx43), whereas connections between astrocytes and other cell types, primarily oligodendrocytes, are mediated by heterotypic gap junctions [19]. Astrocytic Cx43 also forms structures known as hemichannels. Hemichannels are formed when a connexon inserts into the membrane of one cell with no binding partner on another cell, acting as a potential conduit between the intracellular and extracellular space.

In glaucoma, astrocytes in the retina and optic nerve undergo significant changes throughout the course of the disease. Early in the disease they undergo morphological changes to become “reactive” astrocytes, a subset of which can be neurotoxic and amplify RGC death [20,21,22,23,24]. The induction of one neurotoxic subtype is mediated by microglial activation, where inflammatory stimuli cause microglia to release several pro-inflammatory cytokines. These in turn induce a neurotoxic astrocyte phenotype [22]. Reactive astrocytes are key mediators of RGC death in glaucoma [23]. Interestingly, this same process of microglial activation and cytokine release alters the functional properties of astrocytic Cx43. Activated microglia stimulate an increase in astrocytic Cx43 hemichannel activity while simultaneously decreasing gap junctional coupling between astrocytes [25]. The involvement of Cx43 in the glial inflammatory response suggests that changes in Cx43 function may be involved in the early cellular responses to elevated IOP.

Gap junctions and hemichannels have substantially different physiologic and pathologic roles which can impact neurodegenerative disease progression. This review will explore these roles, and we will discuss how astrocytic Cx43 networks may be a valuable target for RGC neuroprotection in glaucoma. We will explore the basic physiologic roles of astrocytic networks, before using knowledge from glaucoma and a variety of other neurologic diseases to build a model of the pro-degenerative and pro-survival influences. We will then highlight the known changes to Cx43 in glaucoma and discuss their consequences. Finally, we will present the currently available tools for selectively targeting Cx43 and evaluate their potential as therapeutic strategies for glaucoma.

## 2. Harnessing Intrinsic Neuroprotective Functions of Astrocytic Cx43 to Prevent Neurodegeneration

Under typical conditions astrocytic gap junctions provide essential functions to maintain homeostasis. Two key astrocyte functions that are in part dependent upon gap junction-mediated networks include the provision of metabolic substrates to neurons and the buffering of the contents of the extracellular space.

### 2.1. The Astrocyte Neuron Lactate Shuttle

Astrocytes are the only significant source of glycogen in the brain. Increased neuronal metabolic demand prompts astrocytes to break down their glycogen stores, undergo glycolysis, and donate lactate to the neurons for energy [26,27,28,29,30,31]. Astrocytic glycogen supports the metabolic demands of both physiological synaptic signaling and action potential propagation, and acts as a reservoir of metabolic substrates when neurons are stressed. A large body of evidence supports the theory that astrocytes are a primary supplier of energy to highly active neurons through the astrocyte neuron lactate shuttle [32]. Astrocytes undergo aerobic glycolysis and produce l-lactate, which is passed to neurons through monocarboxylate transporters. Monocarboxylate transporters are ubiquitously expressed plasma membrane proteins responsible for carrying molecules with one carboxylate group, including the key metabolites lactate and pyruvate, across the cell membrane. The transporters are expressed by both astrocytes and neurons, allowing for the shuttling of lactate from one cell to the other. The release of lactate from astrocytes is positively correlated with neurotransmission, indicating that the release is regulated by neuronal metabolic demand. At excitatory synapses, the uptake of glutamate by astrocytes stimulates glycolysis and lactate production within the astrocytes [33], allowing for delivery of metabolites to supplement the increased metabolic needs of neurons during excitation [34]. Neurons convert this lactate into pyruvate for use in oxidative metabolism, producing abundant adenosine triphosphate (ATP). Astrocytic lactate release appears to be especially important in times of prolonged metabolic demand or transiently decreased metabolic supply from the bloodstream. With prolonged stimulation of neurons there is early oxidative metabolism, which is subsequently sustained over time by activation of the astrocyte neuron lactate shuttle [27]. In many diseases, including glaucoma, neuronal hyperactivity and metabolic dysregulation are early subclinical features [35,36]. Thus, the astrocyte neuron lactate shuttle represents a protective force against these early stressors.

Physiologically, astrocytic lactate supply is vital for various neuronal processes, including plasticity and recovery after stress [37]. The stimulation of lactate release is required for the establishment of long-term potentiation (LTP) at glutamatergic synapses in the hippocampus [38]. The astrocyte neuron lactate shuttle is also important for stressed tissue. If neural white matter tissue is exposed to conditions simulating transient ischemia, astrocytic glycogen stores can sustain axonal function for a period. Depletion of astrocytic glycogen rapidly accelerates the decline in function [39], highlighting an important defense against ischemic conditions.

Interestingly, glycogen is not uniformly distributed throughout the brain [40]. Such regional variability could lead to a topographic imbalance in the size of metabolic reservoir, increasing the vulnerability of certain regions to metabolic stress. However, the astrocytic glycogen stores are not sequestered to just local availability and can be redistributed according to metabolic needs. Cx43 gap junctions allow the nonselective passage of molecules up to ~1–1.2 kilodaltons in size including a number of metabolically significant molecules, notably glucose [41].

Astrocytic metabolic networks play a key role in supporting neural activity. Glucose trafficking through astrocyte gap junctions plays an important role in maintaining hippocampal synaptic activity [26]. Additionally, glycogen stores redistribute over a long distance to support neural function in response to the stress of elevated IOP [35].

The transfer of glucose between astrocytes is driven by a concentration gradient [26], allowing nutrient-rich regions to supply regions with high demand. In a similar manner to the mechanism described in lactate production, neural-activity-mediated regulation of metabolite transfer is reported. For example, astrocytes are suggested to exhibit greater Cx43 expression in co-culture with neurons than in monoculture [16]. Furthermore, excitotoxic treatments triggering neuronal death in vivo lead to a downregulation of connexins in astrocytes located in close proximity to dying neurons [16]. The activity of neurons directly influences astrocytic regulation of Cx43 gap junctions [42].

The mechanism by which this occurs appears to be related to synaptic signaling. Synaptic activity has been directly linked to upregulation of gap junctional communication [43]. Excitatory neuronal transmission increases extracellular glutamate and potassium levels, which in turn enhances gap junctional coupling between astrocytes [44]. Activity-dependent enhancement of coupling is accomplished by post-translational modification of Cx43 channels, increasing the number of active channels within gap junction plaques [43,45]. Such regulation has important physiological consequences. Blocking gap junctions (pharmacologically and genetically), as well as flooding the astrocytic network with metabolically inert D-lactate prevents long-term potentiation (LTP) in mouse cortex, thus eliminating an important component of synaptic plasticity [46]. Local delivery of high levels of L-lactate, the metabolically active enantiomer that can be utilized by neurons, rescues the phenotype and allows for the re-establishment of LTP independent of astrocytic networks [46]. The importance of astrocytic Cx43 for sufficient lactate delivery suggests individual astrocytes are unable to meet the energy demands required for synaptic plasticity and other energetically intensive processes; rather, broad metabolic networks are required to meet such demands. Further, it illustrates that this connectivity is dynamically modulated by neuronal metabolite insufficiency.

As a response to insufficient neuronal metabolites, astrocytes are able to regulate cerebral blood flow and coordinate this regulation broadly though gap junctional networks. Propagation of calcium waves through astrocytic gap junctions controls arteriolar dilation and thus regional blood flow [47,48]. Such coordination is important for maintaining sufficient blood flow, and thus nutrient delivery, in the face of challenges to perfusion. In the eye, targeted miRNA knockdown of glial Cx43 expression compromises the reactivity of both arterioles and venules to challenges to ocular perfusion [49]. Regulation of central nervous system blood flow is a key function and an important mechanism for supporting neuronal metabolism during the changing balance between metabolic supply and demand.

### 2.2. Astrocytic Networks and Extracellular Buffering

Astrocytes tightly regulate extracellular concentration of ions and neurotransmitters used for synaptic transmission. Glutamate and potassium are two important examples of such products whose concentrations are tightly regulated by astrocytes.

Astrocytic glutamate uptake is the main route of glutamate clearance from the excitatory synapse, and the primary mechanism by which the neurotransmitter is recycled [50,51,52]. The uptake of glutamate against its concentration gradient is mediated by sodium-dependent transporters, primarily GLT-1 and GLAST [53]. These proteins take in three Na^+^ and one H^+^ with each molecule of glutamate, while one K^+^ ion is transported outwards [54].

Astrocytes also dynamically modulate extracellular potassium through both active and passive means. Mechanisms for potassium flux include Na/K–ATPase pumps, Na/K/Cl cotransporters, and inwardly-rectifying potassium channels (mainly K_ir_4.1) [55,56]. Astrocytic potassium regulation is essential for regulating synaptic firing and plays a role in modulating neural network firing dynamics [57,58]. Deficiencies in this regulation can lead to aberrant neuronal excitability in the setting of elevated local extracellular potassium concentrations [59].

Astrocytes contribute to the maintenance of the extracellular space through spatial buffering, in which local extracellular increases of a metabolite can be countered by a dispersion to regions of lower concentration [60,61,62]. Gap junctions allow for the expansion of buffering responsibility across a network of cells. Buffering through astrocytic networks increases the capacity for removal of excess K^+^ and glutamate, limiting the accumulation during periods of increased neuronal firing. In the hippocampus, this helps to tone down neuronal excitability. Eliminating these networks through genetic deletion of connexin proteins stresses the buffering capacity of individual astrocytes, leading to cell swelling and decreased uptake [63]. The neuronal consequences of this disconnection are increased excitability, higher presynaptic release probability, and alteration of the postsynaptic receptor composition. However, some coupling-deficient astrocytes may have a large capacity for K^+^ clearance, as measured by the rate of change in extracellular potassium concentrations during stimulation [64]. Still, pathological insults may tip the scale; mice without astrocytic coupling show an increased susceptibility to experimental generation of epileptiform events [64].

The importance of gap junctional coupling extends beyond local ionic redistribution. Neuronal activity causes a depolarization of the astrocyte cell membrane. The electrical coupling of adjacent cells through gap junctions minimizes the magnitude of depolarization in any individual cell, allowing for the efficient maintenance of the electrochemical driving force responsible for potassium and glutamate uptake [65].

An intact astrocytic network allows astrocytes to function more efficiently, regulating local increases in extracellular glutamate and potassium and preventing highly active or diseased neurons from inducing aberrant excitability in neighboring cells.

Neuronal hyperexcitability is implicated in many neurodegenerative pathologies. It is an early feature of Alzheimer’s disease, where it is hypothesized that deficits in astrocytic glutamate buffering may underly the hyperexcitability [66]. Computational modeling suggests that astrocytic spatial buffering can serve a neuroprotective function, mitigating the spreading of pathologic depolarizations [67]. Excitability and dysregulated glutamate signaling are also implicated in diseases ranging from amyotrophic lateral sclerosis [68] and Huntington’s Disease [69] to the degeneration seen in multiple sclerosis [70]. In experimental glaucoma, increased RGC excitability is seen early in the disease [36]. While it is suggested that this could be protective, it more likely represents a preclinical sign of the disease and may be ultimately deleterious and place additional stress on the RGCs [53,71]. Independent of the mechanism driving increased neuronal excitability, an intact astrocytic network likely serves to protect against uncontrolled increases in extracellular glutamate and potassium and prevents the spread of pathologic depolarizations.

A dysregulation of extracellular glutamate and potassium places immense stress on neurons and can be neurotoxic. Astrocytes are essential to the regulation of these molecules, and astrocytic networks provide an additional protective buffer against this stress. Disruption of the network could serve to amplify even small pathologic insults.

## 3. A Role for Astrocytic Cx43 in Neurologic Disease

Gap junctional coupling and hemichannel activity are involved in several diseases affecting the nervous system. Astrocytic Cx43 in particular has been implicated in a wide spectrum of neurologic pathologies including stroke and neurodegeneration. Using evidence from this variety of diseases allows a general model for the roles of Cx43 in neurologic disease to be built.

### 3.1. Diseases of the CNS

Ischemic stroke causes acute metabolic stress to neurons, as they are cut off from the blood supply that brings in a constant supply of nutrients. As discussed previously, astrocytic glycogen transiently maintains neural function in a hypoglycemic setting [39], suggesting that Cx43 may be physiologically important in stroke. Further, neuroprotective reactive astrocytes were first discovered in stroke models, providing a foundation for beneficial astrocyte reactivity during neurodegeneration and stress [72]. Neuroprotective astrocytes activated by ischemic stress are defined by neurotrophic factor release [73,74]. The astrocyte network expands drastically in neuroprotective astrocytes [75] and serves an important role in the neuroprotective phenotype [76].

In patients with unilateral middle cerebral artery ischemic stroke, degenerative damage can be seen in the hippocampus even though it is not served by the artery. In mouse models of ischemic stroke, mice lacking astrocytic Cx43 have a larger volume of tissue damage [77]. They also exhibit increased neuronal apoptosis and markers of inflammation at the site of the lesion [78]. Within ischemic brain tissue there is notable swelling of astrocytic processes at sites of focal ischemia [79,80]. Astrocyte swelling can decouple Cx43 gap junctions and disrupt cell to cell communication [81]. Pharmacologic enhancement of Cx43 gap junctional coupling between astrocytes is protective in stroke models, decreasing the overall volume of the infarcted region [82]. These studies suggest that Cx43 gap junctions are neuroprotective in stroke.

Neurodegenerative disease progresses on a much longer time scale than acute ischemic stroke. However, evidence suggests that Cx43 is still relevant to these diseases. Postmortem tissue from Alzheimer’s Disease patients demonstrates increased immunolabeling of Cx43 at the edges of amyloid plaques [83], which is also seen in brain tissue from many mouse models of the disease [84]. Furthermore, transcriptomic and proteomic analyses of postmortem tissue show an association between upregulation of the *GJA1* gene and worse pathology, as well as increased cognitive deficits, in Alzheimer’s patients [85]. Changes in Cx43 expression have also been seen in two other neurodegenerative diseases, Huntington’s and Parkinson’s, which exhibit astrocytic reactivity and increased Cx43 labeling at the sites of pathology [86,87]. The associations between Cx43 and the pathological and clinical severity of a variety of neurodegenerative diseases suggest a broad role for Cx43 in neurologic disease progression and demonstrate the potential for astrocytic Cx43 to impart both neuroprotective and pro-degenerative influences.

### 3.2. The Pathophysiology of Gap Junctions and Hemichannels

Gap junctions and hemichannels have seemingly opposing pathophysiologic roles. There are many neuroprotective functions of astrocytic gap junctional coupling. In contrast, hemichannels appear to have a pro-degenerative role during pathologic insult. Classically thought to be closed or inactive in a physiologic state, recent evidence supports a role for astrocyte Cx43 hemichannels in mediating processes such as the tuning of glutamatergic signaling through glial neurotransmitter release [88,89]. Nevertheless, prolonged stressors such as ischemia and oxidative stress can cause pathologic hemichannel opening [90]. Their opening in this context is typically deleterious, associated with inflammation, changes in cell membrane potential, and difficulty with osmoregulation [91]. Hemichannels have been implicated as a major cause of tissue swelling post-injury [91].

Inflammation is a prominent feature of numerous diseases, and astrocytes are a key cellular component of the neuroinflammatory response [92]. In general, inflammatory cytokines decrease gap junctional coupling between astrocytes, although there is some evidence that different inflammatory conditions can affect Cx43 differentially, either increasing or decreasing coupling [93,94]. Hemichannels are also a key factor in inflammatory conditions. Proinflammatory cytokines, such as interleukin-1β and tumor necrosis factor-α, decrease the localization of Cx43 to the cell membrane [25]. The administration of lipopolysaccharide, an endotoxin found on Gram-negative bacteria that is frequently used to experimentally induce inflammation, accelerates the degradation of Cx43 proteins in addition to decreasing expression [95]. Alongside this structural change, there is a decrease in intercellular gap junctional communication with a concomitant increase in hemichannel activity [25]. In this context, hemichannels act as a key pro-inflammatory mediator. A major consequence of hemichannel opening is the extracellular release of ATP, which can bind to purinergic receptors and activate the NLRP-3 inflammasome [96], a protein complex that initiates inflammatory cell death. Specifically blocking Cx43 hemichannels decreases overall inflammation and protects against tissue damage [97]. This effect appears to be, in part, due to decreased inflammasome activation [98]. In fact, specific blockade of Cx43 hemichannels is actively being investigated as a potential therapeutic for many neurologic and non-neurologic diseases [96,97,99,100,101,102,103,104,105].

It is less clear whether Cx43 gap junctional coupling is exclusively protective or maladaptive in pathological conditions. From a metabolic perspective, the redistribution of resources through the gap junctional network seems to be neuroprotective. From a buffering perspective, the network allows astrocytes to regulate the extracellular environment more effectively. However, because of the nonselective nature of these gap junctions there is also the potential that they mediate the spread of damaging cues. In one study, overexpression of Cx43 in injury-resistant glioma cells left the cells more vulnerable to damage, as they were increasingly coupled to more vulnerable cells [106]. There was an associated increase in the spread of calcium waves through the cells. Calcium waves are the primary means of astrocytic signaling, in the absence of an excitable membrane, and coordinate important physiologic processes such as glial neurotransmitter release [107]. However, excessive and dysregulated intracellular calcium signaling can activate pro-apoptotic pathways [108]. Gap junctional networks could mediate excessive calcium spread from astrocyte to astrocyte, acting as a conduit for the propagation of disease. The Cx43 overexpression leading to death of neighboring glioma cells may have been a cell-type specific effect, however. In studies involving a variety of animal models of neurologic diseases, increasing gap junctional coupling is associated with decreased neuronal death and improved histologic measures [82,109].

### 3.3. A General Model for Astrocytic Networks in Neurodegenerative Disease

**Figure 2:**
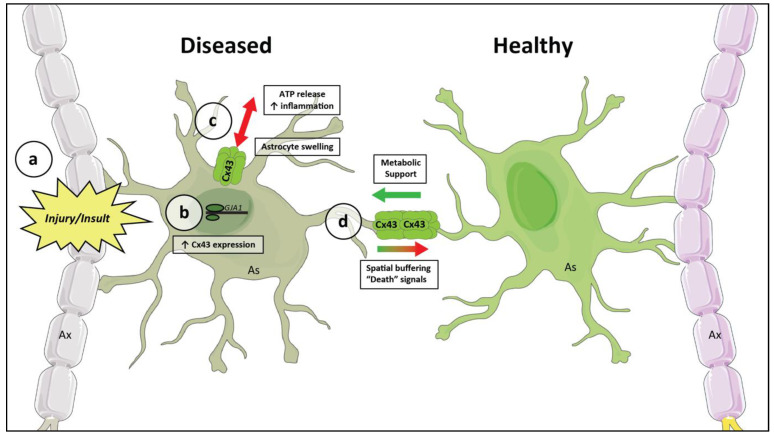
A working model of the role of Cx43 in neurologic disease. A pathologic insult to neural tissue (**a**) causes an increased expression of Cx43 (**b**). Cx43 connexons are assembled and transported to the cell membrane, increasing the number of unopposed hemichannels (**c**). This allows for the extracellular release of adenosine triphosphate (ATP), causing increased inflammation and microglial activation. Open Cx43 hemichannels also cause cellular swelling. Increased degradation and post-translational modifications, as well as physical swelling of the cell, cause a decrease in Cx43 gap junctional coupling (**d**). Consequently, there is less available metabolic support from distant astrocytic glycogen stores. There is also a decreased ability to spread out the effect of local increases in extracellular glutamate and potassium through spatial buffering. However, this may also be protective by limiting the spread of calcium waves and other “death signals” to nearby healthy tissue. Cells on the left represent a generic region of CNS pathology, while healthy brain tissue is on the right. Ax, axon; As, astrocyte; and Cx43, connexin-43.

Figure 2 demonstrates a basic working model of the current understanding. Hemichannels are largely maladaptive, increasing regional inflammation and leading to tissue swelling and cell death. Gap junctions, on the other hand, may be both protective and harmful by facilitating metabolic support for stressed neurons and increasing buffering capacity, yet also maladaptive by allowing for the spread of “death signals.”

## 4. Changes to Cx43 in Glaucoma and Other Optic Neuropathies

The initial site of stress conveyed to axons by elevated intraocular pressure is at the optic nerve head (ONH) [110], the portion of the optic nerve where the unmyelinated RGC axons pass through the sclera. This region of the nerve is densely populated with astrocytes, which offer structural and physiologic support to the axons and are involved in early responses to the stress of elevated IOP. Astrocytes are also present in significant quantities throughout the inner retina. In both humans and rodents there are astrocytic networks localized to the ganglion cell layer and the nerve fiber layer, which see significant degenerative changes in severe glaucoma. Immunolabeling shows that these are continuous with the optic nerve astrocytes [111,112].

Astrocytes in the retina express both Cx43 and Cx30. Cx30 is localized primarily to astrocytic endfeet near blood vessels whereas Cx43 is more diffusely distributed along the astrocyte [111,112]. There is also some expression of Cx43 in retinal Müller glia, though this varies by animal model and occurs at significantly lower levels than in astrocytes [113]. Within the optic nerves of humans and rodents, particularly in the ONH, there is dense immunolabeling of Cx43 localized to astrocytes [8,9,113].

A densely connected astrocytic network may be of particular physiologic importance for the ONH portion of the nerve. The axons in this region are unmyelinated. Without myelination, axons have decreased signaling efficiency and much higher metabolic demand [114]. Astrocytic Cx43-mediated networks may be important for supporting the demand of this region and serve as a defense against pathologic stress. Alterations in the function of this network could increase the susceptibility of this key visual pathway to stress and injury.

### 4.1. Ischemic and Traumatic Optic Neuropathies

Cx43 gap junctions and hemichannels are involved in the progression of damage to the optic nerve in multiple disease models. In an ex vivo model of optic nerve ischemia there was a notable upregulation of astrocytic Cx43 expression following onset of ischemia, which continued to rise before peaking at 3 days [115]. Transient downregulation of Cx43 expression with specific antisense oligodeoxynucleotide reduced nerve swelling and cell death, and decreased the degree of astrocyte reactivity and microglial activation. This knockdown was not specific to hemichannels or gap junctions, but nonetheless prevented the injury-associated Cx43 upregulation, presumably reducing the formation of new unopposed hemichannels [25]. A more focal injury, caused by partial optic nerve transection, created a biphasic expression pattern of connexins [116]. There was an early increase in astrocytic Cx43 labeling at site of injury, which then decreased at the initial site but increased in neighboring regions as the damage progressed. Interestingly, there was also greater labeling in the region of retina where the RGCs whose axons passed through the site of optic nerve injury were located. These findings demonstrate that changes to astrocytic Cx43 are intimately involved in the response to injury in the optic nerve, and that modulating Cx43 hemichannels and gap junctions can alter the progression of damage. The changes are initially focal, localizing to the size of injury, but over time spread spatially, even well beyond the site of injury into the retina.

### 4.2. Changes to Cx43 in Glaucoma

Astrocytic networks are also important in the pathophysiology of glaucomatous optic neuropathy. Glial activation in the retina and optic nerve is an early and persistent feature of glaucoma [117]. Real time PCR comparing primary cultures of astrocytes from normal and glaucomatous human eyes indicates that expression of *GJA1* is higher in glaucomatous astrocytes compared to normal age-matched controls [118]. Structurally, changes in immunolabeling of Cx43 on the optic nerve head have been demonstrated in a study of post-mortem eyes of glaucoma patients [111]. There was a significant increase in Cx43 on astrocytes within the lamina cribrosa, at the site of mechanical stress to the ONH. In the retina there was an increase in both the intensity and amount of Cx43 labeling in the ganglion cell layer. These patterns parallel those seen in animal models of optic nerve injury, although they reflect changes over a longer time scale.

The mechanism of injury in glaucomatous neurodegeneration involves an increased genetic and/or environmental susceptibility to elevated IOP at the optic nerve head. Exposing primary human ONH astrocytes to elevated hydrostatic pressure causes a decrease in intercellular communication and a redistribution of Cx43 away from the cell membrane [119]. The remaining gap junctions on the membrane are also more highly phosphorylated. Phosphorylation on several serine and tyrosine residues in the C-terminal region of the Cx43 protein can dramatically decrease the permeability of the gap junction [120,121]. Thus, even when there is increased Cx43 expression, as seen in glaucomatous tissue, there is a decrease in the permeability of gap junctions. As seen in other models of neurologic disease, astrocytes in glaucomatous optic nerves have increased expression of Cx43, which leads to increased hemichannel activity, but paradoxically there is a decrease in gap junctional communication.

#### 4.2.1. Physiologic Consequences

The structural changes in Cx43 distribution have important functional implications for the progression of glaucoma. Though astrocytic Cx43 networks are protective against metabolic stress to an extent, astrocytes lose their gap-junctional coupling when deprived of glucose for longer periods of time [122]. Chronic metabolic deprivation also increases hemichannel permeability [123]. Axons in the glaucomatous optic nerve are energy depleted and exhibit signs of chronic metabolic stress, marked by decreased available glucose and lower expression of the monocarboxylate transporters involved in the astrocyte neuron lactate shuttle [124].

In the microbead occlusion mouse model of glaucoma, elevated IOP in one eye causes the mobilization of glycogen stores from the unaffected eye to the damaged eye [35]. This redistribution improves axon function and visual function in the acceptor eye but leaves the donor eye vulnerable to subsequent damage. Genetically knocking out Cx43 in these mice eliminates this transfer of resources. This evidence emphasizes both the neuroprotective and maladaptive potential of astrocytic networks in the context of degenerative disease.

Alongside metabolic changes, oxidative stress is a key factor in the pathophysiology of glaucoma [125]. It is also a regulator of gap junctional coupling. In other cells, like osteocytes, high levels of oxidative stress tend to decrease coupling and increase hemichannel activity [126]. Increasing Cx43-mediated coupling in cultured cells is protective against hydrogen-peroxide-induced cytotoxicity, suggesting that it helps mitigate the damage caused by oxidative stress [126,127]. The changes to astrocytic coupling caused by glaucoma may be part of a vicious cycle that causes further metabolic stress, as the injured regions are cut off from distant glycogen supply. It also likely decreases the ability of astrocytes to defend against cytotoxic damage. However, this may serve to isolate an injured region, preventing the further spread of damage to other sites.

#### 4.2.2. Gap Junctions and the Spread of Pathology

Studies of unilateral optic nerve damage offer an insight into the role of gap junctional networks into the spread of pathology. Animal studies demonstrate that unilateral trauma results in changes to the eye contralateral to the injury, with marked alterations to retinal astrocytes, microglia, and ganglion cell populations [128,129,130,131,132]. The response in the contralateral eye is weaker than in the eye ipsilateral to the injury, but still significantly greater than control tissue from uninjured animals. Astrocytes in the contralateral retinal ganglion cell and nerve fiber layers take on a reactive phenotype, characterized by cellular hypertrophy and increased labeling of glial fibrillary acidic protein (GFAP) [133]. They also express greater levels of Cx43, raising the possibility that this spread of damage could be mediated by astrocytic networks. However, the evidence is not conclusive and merits further investigation. Genetic or pharmacologic elimination of Cx43 could illuminate whether these networks potentiate or mitigate propagation.

There is strong evidence that glaucoma involves changes in astrocytic Cx43 in the retina and optic nerve. Its role appears to be complex, with hemichannels and gap junctions having important functional distinctions. The precise role of gap junctional networks, whether protective or maladaptive, is incompletely understood and merits further investigation. Proper tools are needed to dissect out the precise physiologic changes seen during disease progression.

## 5. Pharmacologic Tools for Modulating Cx43 Networks

### 5.1. Experimental Drugs

Historically, several drugs with potent actions on gap junctional communication have been used as tools for studying gap junctional networks. Carbenoxolone, a glycyrrhetinic acid derivative that comes from licorice, causes a potent but nonspecific inhibition of gap junctional coupling [134]. It also nonspecifically blocks connexin hemichannels [135], making it a useful and commonly-used tool for the study of connexin physiology. However, there are some limitations to its usage. Significantly, its use in ex vivo and in vivo studies should be avoided as it has a direct impact on neuronal signaling, independent of its action on connexins [136]. There is also limited translatability, as the drug has significant adverse effects [137].

Another class of drug often used to experimentally modulate gap junctional signaling are quinine and other derivatives of *Chinchona* tree bark. These drugs have been used since at least the early 20th century for a variety of conditions [138], most frequently in modern times as antimalarials [139,140]. These drugs also block coupling in a nonspecific manner [141,142]. One such drug in particular, mefloquine, acts on Cx43 [142,143]. However, the effects on gap junctions only occur at high concentrations, limiting therapeutic utility.

### 5.2. Cx43-Specific Targeting

**Figure 3:**
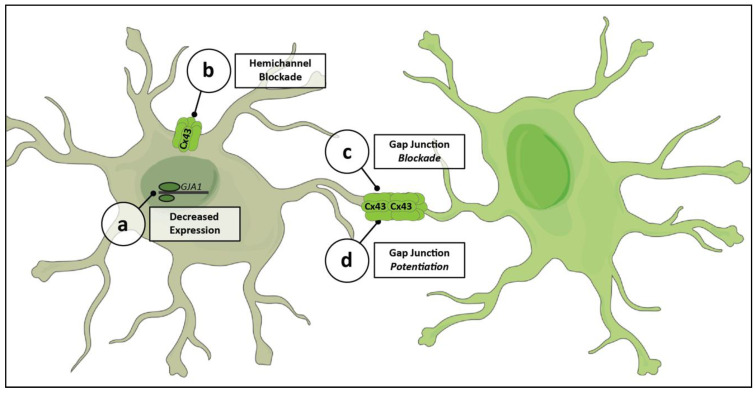
Mechanisms for targeting Cx43 mediated astrocytic networks. Modulation of Cx43 physiology can occur at multiple levels. Substances may decrease the level of expression of Cx43 (**a**), limiting the production and assembly of both gap junctions and hemichannels. Others may directly target one of these functional structures. Some seek to decrease hemichannel activity by selectively blocking Cx43 hemichannels (**b**). Often, agents may directly decrease the degree of gap junctional coupling between adjacent cells (**c**). Other recent drugs have sought to increase the degree of coupling at Cx43 gap junctions (**d**). Cx43, connexin-43; GJA1, gene encoding Cx43, gap junction alpha-1.

Recently, tools have been developed to target more specific aspects of astrocytic Cx43 physiology. The targeted mechanisms of action of these substances allow for studies to be more precise in their probing of the significance of astrocytic networks in physiologic and pathologic processes. They also represent potential pathways for the development of targeted therapies. Figure 3 shows an overview of the various processes that can be acted on by these agents.

One of the first Cx43-specific interventions developed was the use of antisense oligodeoxynucleotides (asODN) to decrease the expression of Cx43 (Figure 3a) [115]. asODN are short chains of nucleotides that can selectively decrease the expression of a gene by binding to mRNA and preventing its translation. This allows for the targeting of a specific connexin, in a transient manner and with few off-target effects. asODN are able to significantly improve disease outcomes in models of optic nerve ischemia [115] and spinal cord injury [144]. However, they are limited by their inability to differentially act on gap junctions and hemichannels, as they blindly inhibit all Cx43 RNA translation.

An additional strategy for modulating Cx43 physiology is the use of mimetic peptides. These are short peptide sequences designed to match portions of the Cx43 protein, which can bind to specific sites and interfere with functioning. The goal of developing these tools is to be able to specifically alter Cx43 hemichannel activity while not affecting gap junctions. A number of these peptides have been developed, with varying efficacies. Peptide5, made against the extracellular regions of connexin-43, can selectively block hemichannel activity (Figure 3b) when used at a low concentration [103,145]. Higher concentrations of the peptide, though, also interfere with gap junctional coupling (Figure 3c) [145]. Administration of peptide5 is protective in a number of neurologic disease models, including retinal ischemia and spinal cord injury [146,147]. Additional mimetic peptides have been developed attempting to improve the specificity of targeting to hemichannels [97,148,149].

An alternate strategy to countering the negative impact of Cx43 hemichannels is the enhancement of protective gap junctional coupling (Figure 3d). Danegaptide, a small peptide drug in development, maintains Cx43 gap junctional coupling during stressed conditions, improving cell to cell communication [82,150,151]. It is thought to work by modifying Cx43 in order to reduce gap junction closing and prevent intercellular uncoupling [150]. This is selective to gap junctions, as there is no effect on hemichannel activity [82]. Danegaptide’s enhancement of gap junctions is protective in a variety of models, including diabetic retinopathy and ischemia-reperfusion brain injury [82,109].

These are powerful tools that could be used to probe the specific physiologic roles of Cx43 in disease and could lead the way for the development of targeted therapies that act on astrocytic networks.

## 6. Conclusions

While Cx43 is a promising therapeutic target for a disease in need of new treatment options, further studies are needed to evaluate the complex role of Cx43-mediated astrocytic networks in glaucomatous neurodegeneration. A leading challenge is understanding the link between changes in protein expression and functional measures. Increased Cx43 expression, in the context of disease, is likely linked to increased hemichannel activity and not gap junctional coupling. However, many studies do not evaluate this in depth. While functional measures are not a viable option for human patient samples, there are good rodent models of glaucoma that should be evaluated in more detail. These distinctions are especially important given the seemingly opposing roles of the two Cx43 structures. Hemichannels are key modulators of inflammation, and blocking their activity has a neuroprotective effect. On the other hand, gap junctional networks may be both protective and deleterious. Connections between injured and healthy regions could allow for metabolic support of stressed neurons, but also serve as a pathway for the spread of neurodegenerative stress or a siphon of metabolic resources. The relative importance of these differential functions could also change over time, as glaucoma is a chronic and slowly progressing disease. Perhaps gap junctional communication is protective early on, but as metabolic resources are depleted it facilitates pathologic spread. Recently developed tools for targeted modulation of Cx43 will allow for a careful exploration of the specific physiologic and pathophysiologic impact of astrocytic networks. Further, the ability to specifically target the pathophysiologic roles of Cx43 will allow for more effective disease modification and help limit off-target effects, given the broad importance of gap junctions across the CNS. With an improved understanding of the complex role of astrocytic Cx43 in glaucoma and other neurodegenerative diseases, there is the potential for immense therapeutic benefit.
